# Supporting Self-Management in Bipolar Disorder: Mixed-Methods Knowledge Translation Study

**DOI:** 10.2196/13493

**Published:** 2019-04-15

**Authors:** Erin E Michalak, Emma Morton, Steven J Barnes, Rachelle Hole, CREST.BD, Greg Murray

**Affiliations:** 1 Department of Psychiatry University of British Columbia Vancouver, BC Canada; 2 Centre for Mental Health Swinburne University of Technology Melbourne Australia; 3 Department of Psychology University of British Columbia Vancouver, BC Canada; 4 School of Social Work University of British Columbia Okanagan, BC Canada

**Keywords:** bipolar disorder, eHealth, self-management, knowledge translation, community-based participatory research, mHealth

## Abstract

**Background:**

Self-management is increasingly recognized as an important method through which individuals with bipolar disorder (BD) may cope with symptoms and improve quality of life. Digital health technologies have strong potential as a method to support the application of evidence-informed self-management strategies in BD. Little is known, however, about how to most effectively maximize user engagement with digital platforms.

**Objective:**

The aims of this study were (1) to create an innovative Web-based Bipolar Wellness Centre, (2) to conduct a mixed-methods (ie, quantitative and qualitative) evaluation to assess the impact of different sorts of engagement (ie, knowledge translation [KT]), and (3) to support engagement with the self-management information in the Bipolar Wellness Centre.

**Methods:**

The project was implemented in 2 phases. In phase 1, community-based participatory research and user-centered design methods were used to develop a website (Bipolar Wellness Centre) housing evidence-informed tools and strategies for self-management of BD. In phase 2, a mixed-methods evaluation was conducted to explore the potential impact of 4 KT strategies (Web-based webinars, Web-based videos, Web-based one-to-one *Living Library* peer support, and in-person workshops). Quantitative assessments occurred at 2 time points—preintervention and 3 weeks postintervention. Purposive sampling was used to recruit a subsample of participants for the qualitative interviews, ensuring each KT modality was represented, and interviews occurred approximately 3 weeks postintervention.

**Results:**

A total of 94 participants were included in the quantitative analysis. Responses to evaluative questions about engagement were broadly positive. When averaged across the 4 KT strategies, significant improvements were observed on the Bipolar Recovery Questionnaire (*F*_1,77_=5.887; *P*=.02) and Quality of Life in Bipolar Disorder (*F*_1,77_=8.212; *P*=.005). Nonsignificant improvements in positive affect and negative affect were also observed. The sole difference that emerged between KT strategies related to the Chronic Disease Self-Efficacy measure, which decreased after participation in the webinar and video arms but increased after the Living Library and workshop arms. A subsample of 43 participants was included in the qualitative analyses, with the majority of participants describing positive experiences with the 4 KT strategies; peer contact was emphasized as a benefit across all strategies. Infrequent negative experiences were reported in relation to the webinar and video strategies, and included technical difficulties, the academic tone of webinars, and feeling unable to relate to the actor in the videos.

**Conclusions:**

This study adds incremental evidence to a growing literature that suggests digital health technologies can provide effective support for self-management for people with BD. The finding that KT strategies could differentially impact chronic disease self-efficacy (hypothesized as being a product of differences in degree of peer contact) warrants further exploration. Implications of the findings for the development of evidence-informed apps for BD are discussed in this paper.

## Introduction

### Background

The tremendous impact of mental disorders—for the people who live with them, their families, and society—is well established. Mental health conditions account for 14% of the global burden of diseases worldwide and 37% of all healthy life years lost from chronic diseases [1]. Millions of people worldwide are suffering and losing quality of life (QoL) due to mental disorders, and many will lose their lives. Bipolar disorder (BD)—a mood disorder characterized by pronounced variability in mood, activity, and energy levels [2]—contributes significantly to this global burden of disease. Robust epidemiological studies indicate a 1% to 2% lifetime prevalence for BD [3], and it is estimated that 6% to 10% of people with this condition will die by suicide [4]. However, these “misery statistics” [5] do not tell the whole story; people with BD can and do experience good health and QoL [6-8].

Effective support for self-management—“the plans and/or routines that a person with BD uses to promote health and QoL” [9]—is viewed as 1 important route to optimizing outcomes in BD. Our Collaborative Research Team to study psychosocial issues in Bipolar Disorder (CREST.BD) has spent the past decade incrementally advancing the literature on self-management and BD. Outputs have included qualitative explorations of self-management application in BD [7,8,10], a Delphi Consensus Consultation project [9], and a review of electronic health/mobile health (mHealth) apps for self-management of the condition [11]. These findings complement a solid international literature speaking to the importance of self-management in terms of impacting health and QoL in people with BD and recommendations for supported self-management in international BD clinical treatment guidelines (eg, [12]). However, as recently noted, “means to support these self-management interventions...are critically needed” [13].

Digital health technologies offer clear potential as 1 route to support the application of evidence-informed self-management strategies in BD, and there is no doubt that people with the condition are seeking health information on the Web. An international survey of 1222 people with a diagnosis of BD from 17 countries reported that 81% of respondents described themselves as internet users; 78% of this sample sought Web-based information related to their diagnosis [14,15]. The most commonly cited reasons for internet searching were to seek more information on the symptoms of BD, prescription drug information, the general course of illness, coping strategies, and medication side effects. Notably, 67% of respondents said that seeking Web-based information helped them to cope with their BD “sometimes or frequently.” Respondents were more likely to report a positive impact of Web-based information on their ability to cope if they were “always able to find what they were looking for” [14]. Our own research into information seeking on the Web in youth with BD has indicated that what youth “are looking for” is credible, safe, and stigma-free Web-based information about their condition [16]. However, there are prevailing concerns about the quality and accuracy of health information on the Web [17,18].

Taken together, the key messages from emerging research in the BD digital technology arena are 2-fold. First, it is clear that people with BD are looking for Web-based information and support to optimally manage their condition. Accordingly, BD research and clinical communities need to rise to the challenge of providing high-quality, evidence-informed Web-based information and resources to meet this need. Second, we need to advance our understanding of how to best maximize user engagement with the Web-based tools we produce. This mixed-methods study was designed to address these needs by assessing the impact of a range of digital and face-to-face engagement (ie, knowledge translation [KT]) strategies on user engagement and health-related outcomes in Canadians with BD developed for a Bipolar Wellness Centre website.

### This Study

The aim of this project was to advance understanding of KT strategies in BD. To this end, we investigate 4 distinct strategies for encouraging engagement with a Web-based BD resource. The strategies were all face-valid methods for engaging people with lived experience with health information but differed in terms of resource intensiveness and accessibility. The 4 strategies were Web-based webinars, Web-based videos, Web-based one-to-one *Living Library* peer support, and in-person workshops. The Web resource to which these KT strategies related (the Bipolar Wellness Centre) was a newly developed website structured around our group’s approach to the domains of QoL in BD. Funding from the Canadian government in the form of a 2-year Canadian Institutes of Health Research (CIHR) “Knowledge to Action” grant supported the development of the central website and the accompanying mixed-methods investigation of how different KT strategies impacted engagement with the content held within it. The research project is positioned as the initial phase of a longer-term developmental program of research to advance understanding of the potential utility of digital health technologies for supporting self-management in people with BD.

## Methods

### Overview

The project was conducted under the auspices of CREST.BD [19], a Canadian-based network dedicated to collaborative research and KT in BD. CREST.BD specializes in community-based participatory research (CBPR), where researchers and knowledge users (in this case, people with BD and BD health care providers) work hand-in-hand [20]. Informed by a decade of research and integrated KT, CREST.BD has developed a specific model of CBPR for BD [21] and dedicated Web and social media platforms [22].

The project was implemented across 2 study phases over a 2-year period. In the first phase, we applied the principles of integrated KT, CBPR, and user-centered design to develop a website (the Bipolar Wellness Centre, described below) to house evidence and tools on self-management strategies for BD. In the second phase, we conducted a mixed-methods (quantitative and qualitative) evaluation of the impact of 4 bespoke (Web-based or in-person) KT strategies designed to foster engagement with the content housed within the Bipolar Wellness Centre. The primary aims of the quantitative study component were to assess whether the 4 KT strategies (1) represented effective engagement strategies and (2) impacted health-related outcomes in study participants with BD. The aim of the qualitative study component were to explore whether the 4 KT strategies represented effective engagement strategies. Participants in the study self-selected 1 of the 4 KT strategies in which to participate.

### Development of the Bipolar Wellness Centre

During the first phase of the study, we developed, within an integrated KT and CBPR framework, our signature website, CREST.BD “Bipolar Wellness Centre” [23]. The primary goal of the Bipolar Wellness Centre is to support self-management in people with BD and empower them to manage and improve their health and QoL. Structurally, the website’s design emulates our theoretical understanding of QoL in BD, developed on the basis of our longstanding program of research exploring the QoL construct in BD (eg, [6,24]). Self-management categories were presented according to the 14 domains of QoL measured by our Quality of Life in Bipolar Disorder (QoL.BD) scale [25] and its Web-based counterpart, the QoL Tool [26], which assesses the cardinal life areas impacted by BD (eg, mood, sleep, physical health, and cognition), more concrete life areas (eg, finances, home, work, education, and leisure), and some which are more psychosocially orientated (relationships, self-esteem, spirituality, identity, and independence).

In keeping with the principles of CBPR, we fused the expertise of academic researchers, people with BD, and health care providers to cocreate the content in the Bipolar Wellness Centre. First, we conducted 3 consultation events in partnership with the Mood Disorders Association of Ontario (MDAO) in the Canadian cities of Toronto, Bowmanville, and Guelph, to develop the “look and feel” of the website. These consultations with people with BD resulted in the selection of an overarching nautical metaphor (effective self-management of BD has been likened to “to a ship that’s always righting itself” [7]). Identification of the research evidence and tools for the Bipolar Wellness Centre was a thoroughgoing process. To determine the content for each of the 14 self-management areas, we first appointed a CREST.BD lead and then conducted academic and grey literature reviews. Draft section content was created, revised by a plain language writer, and then reviewed by the CREST.BD Community Advisory Group. Our early design consultations also prioritized accessibility of website content for people with BD who were struggling with symptoms or cognition problems. The research evidence was, therefore, presented in succinct text form and audio version and augmented by brief “key messages” and ways to “take action.” Also provided are key scientific references and carefully curated (and annually updated) area-specific resources and tools (eg, evidence-informed tools to support the application of self-management strategies for sleep).

**Figure 1:**
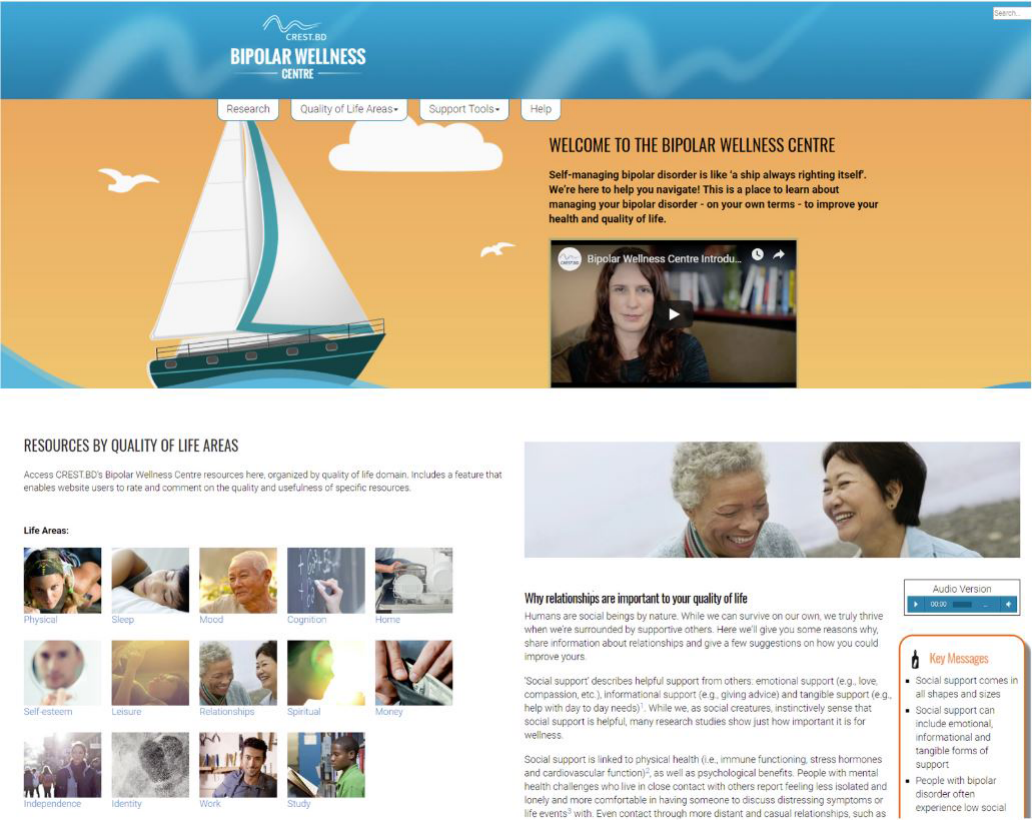
Bipolar Wellness Centre.

The official launch of the Bipolar Wellness Centre (see Figure 1) occurred on World Bipolar Day (March 30, 2015). The project to evaluate the impact of the 4 KT strategies (ie, webinars, videos, Living Library, and workshops, described in full below) was conducted throughout 2015. After the evaluation of the KT strategies was complete, the webinars and the videos (but not the Living Library nor workshop strategies) were made publicly available through the website (funding parameters did not permit implementation or longer-term sustainability of the Living Library or workshop strategies).

### Development of and Evaluation Methods for the Bipolar Wellness Centre Knowledge Translation Strategies

**Figure 2:**
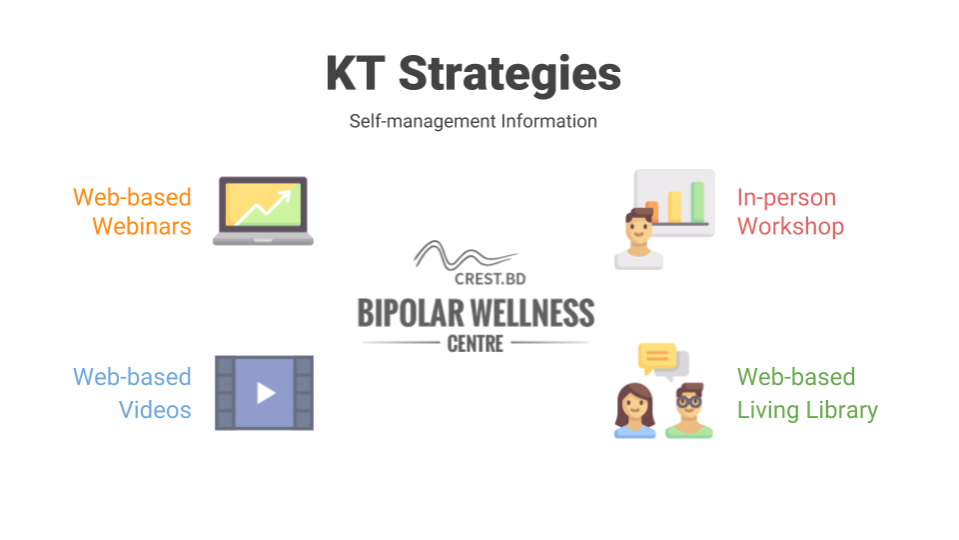
Overview of knowledge translation strategies. CREST.BD: Collaborative Research Team to study psychosocial issues in Bipolar Disorder; KT: knowledge translation.

During the second phase of the study, we developed (again within an integrated KT and CBPR framework) and evaluated 4 bespoke Bipolar Wellness Centre KT strategies (see Figure 2).

#### Web-Based Webinars

**Figure 3:**
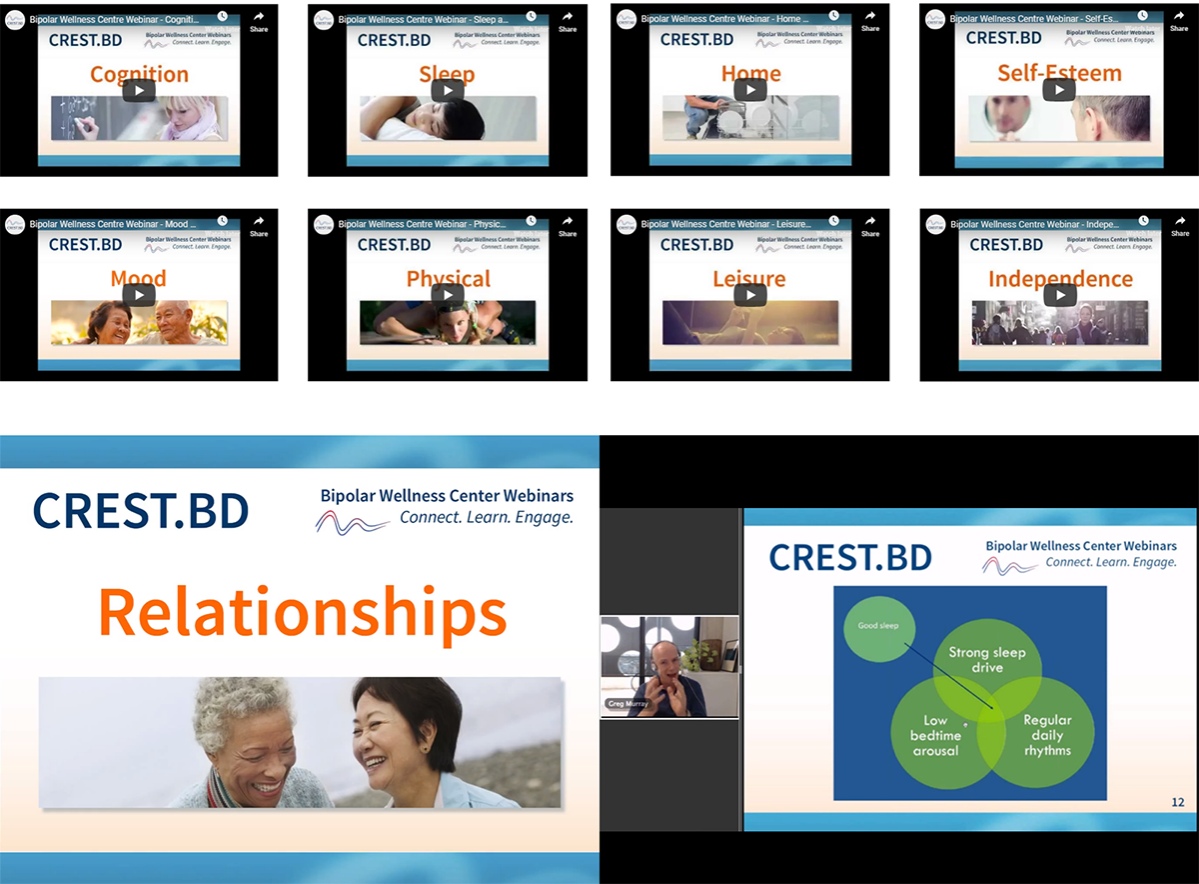
Webinars.

A total of 14 webinars [27] (see Figure 3) were produced, 1 corresponding to each self-management area; each webinar was delivered by a CREST.BD member with internationally recognized expertise in the focal self-management area. Overall, 12 of the webinar presenters were academics (one of whom, coauthor SJB, also has BD) and 2 presenters provided lived experience of BD. Webinars were 15 to 20 min in length (not including questions and answers [Q&A]) and each followed a structured delivery format, covering (1) education about the specific self-management area (eg, the importance of sleep as a self-management strategy and its relation to QoL, as summarized in the text section relating to sleep and BD on the Bipolar Wellness Centre); (2) some key messages from the current research evidence in that area (eg, as provided in the text and literature on the Bipolar Wellness Centre); and (3) how to take action (eg, recommendations for tools to support self-management strategies for sleep, as housed on the Bipolar Wellness Centre). The webinars (split screen of video of presenter and PowerPoint slides) were prerecorded to ensure audio quality and delivery fidelity. The actual webinars were, however, delivered “live” (ie, the prerecorded webinar with a live Q&A with the presenter) to research participants. Following evaluation, a recording of the webinar and Q&A was made publicly available through the website.

#### Web-Based Videos

**Figure 4:**
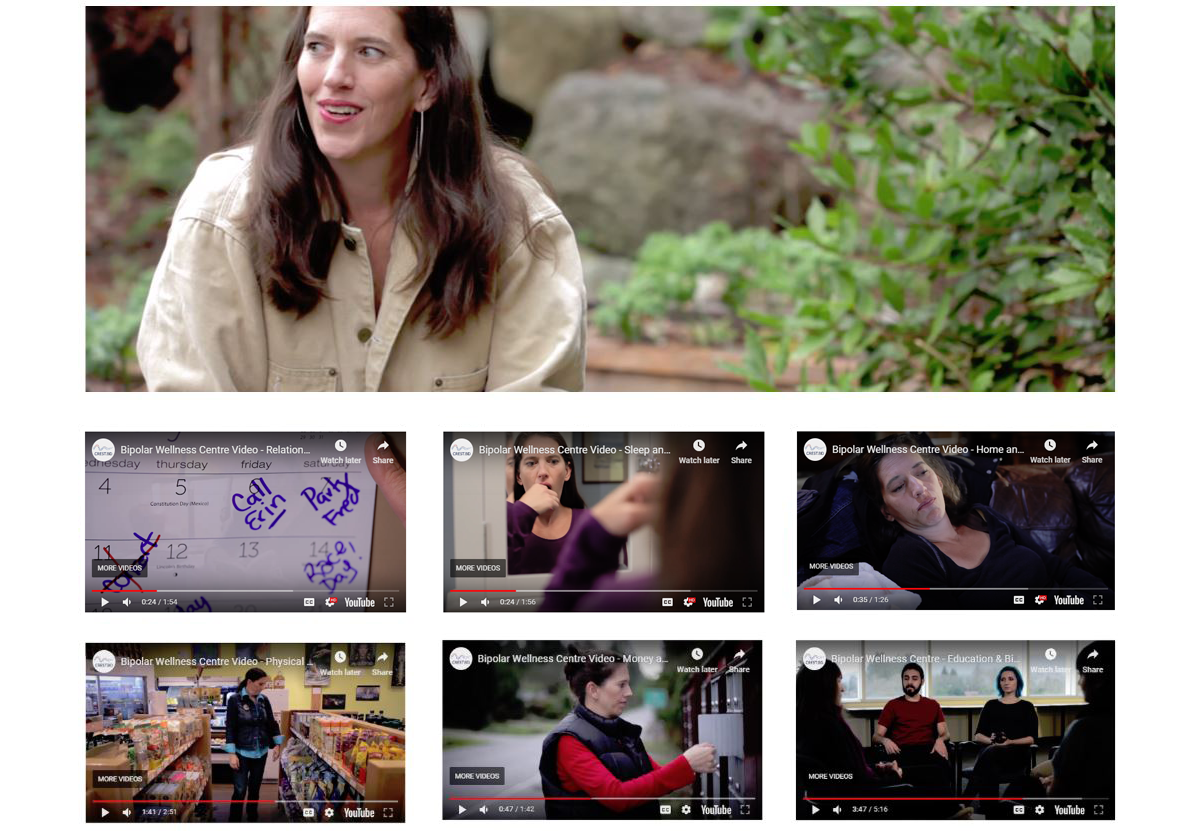
Videos.

Overall, 6 videos [28] (see Figure 4) were developed in collaboration (with the production company *As You Like It*) in the areas of mood, sleep, relationships, physical health, finances, and home life. Videos were 2 to 4 min in length and all featured CREST.BD member Victoria Maxwell, an actor and mental health educator who lives well with BD. Video content (development of which was informed by the self-management area content on the Bipolar Wellness Centre) was similarly structured across the 6 videos, with the actor or narrator introducing the key self-management area, followed by pragmatic examples of self-management behaviors in everyday life (eg, how to apply the principles of good sleep hygiene).

#### Web-Based Living Library

**Figure 5:**
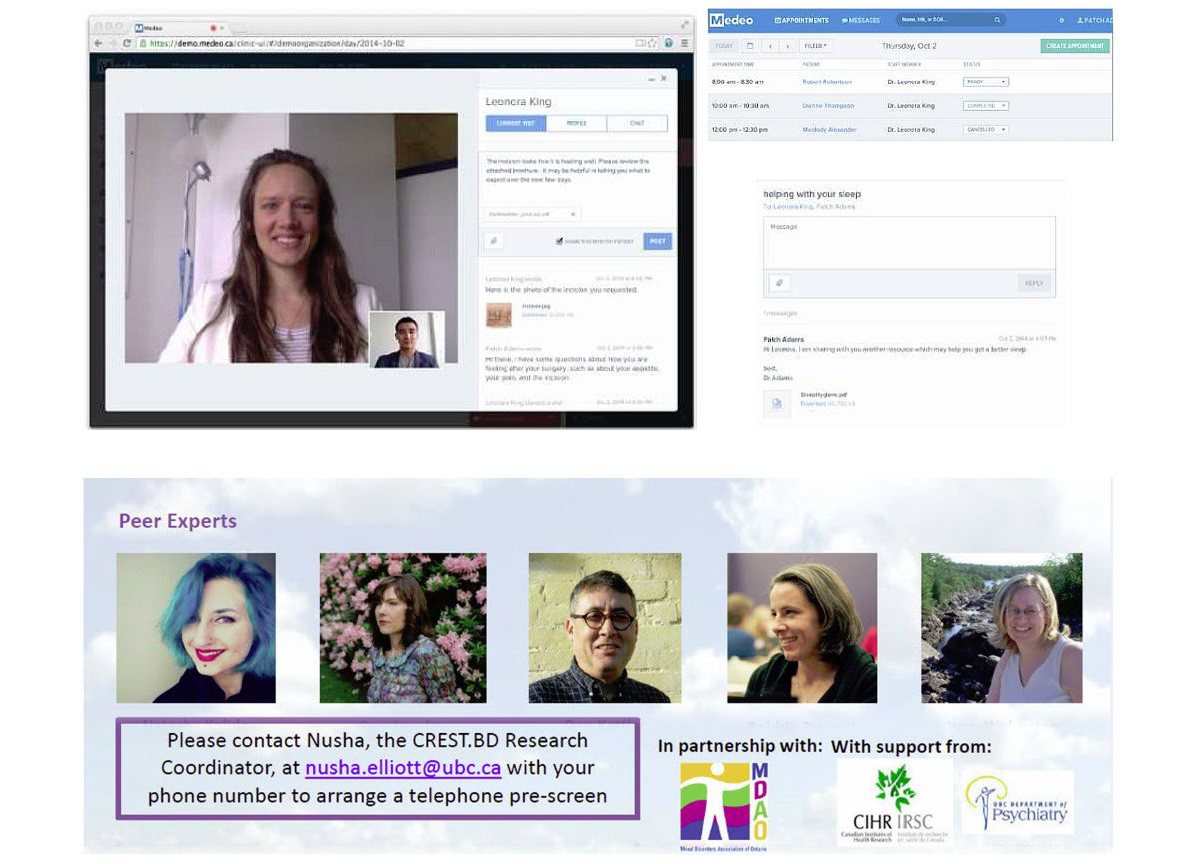
Living Library.

The Living Library KT strategy (Figure 5) consisted of a single 45-min session between the research participant and a Living Library “expert” (ie, 1 of 5 experienced peer-researchers living well with BD who had undergone thorough training in Bipolar Wellness Centre content and navigation) via a secure Web-based telehealth system (Medeo). Sessions were relatively unstructured, but the Living Library experts were instructed to explore the research participant’s priorities for self-management focus and to navigate the participant through the germane content areas and tools in the Bipolar Wellness Centre (with the exception of the videos and webinars, which were not, at that time, made available via the website).

#### In-Person Workshops

**Figure 6:**
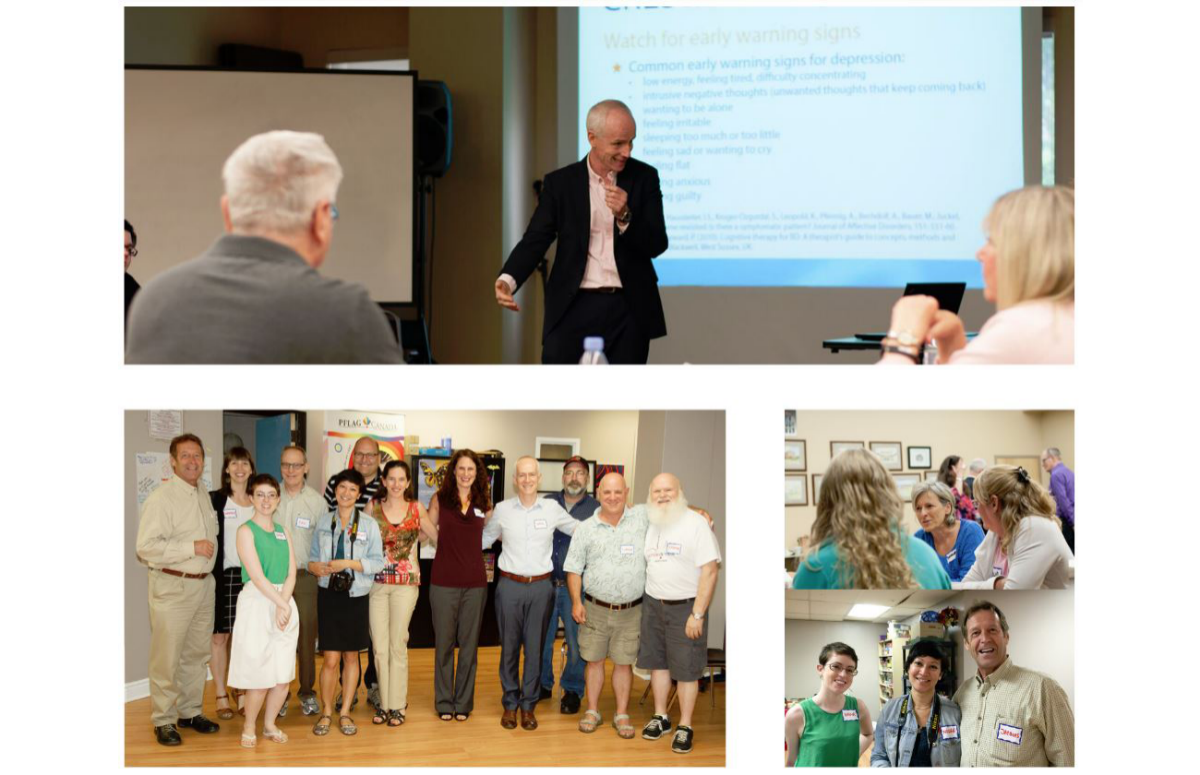
Workshops.

A structured 2.5-hour in-person group workshop (Figure 6) was developed focused on 2 self-management domains (mood and sleep, cardinal BD self-management areas). Workshops were codelivered by 3 CREST.BD team members (coauthors EEM and GM and Victoria Maxwell) and provided structured delivery of didactic, role-play, and small group work. They were delivered 3 times in the Canadian cities of Ottawa, Kingston, and Toronto (sample size at each workshop was 6, 10, and 16, respectively) in partnership with MDAO.

### Recruitment

To be eligible, participants were required to be (1) aged 19 years or above, (2) able to communicate in English, (3) able to provide informed consent, (4) a resident of Canada, and (5) have a self-reported diagnosis of BD (Type I, II, or not otherwise specified). Participants were recruited via diverse methods including CREST.BD face-to-face (eg, outreach at community events and conferences), Web-based (eg, news releases on the CREST.BD website and blog), and social media communications (eg, CREST.BD Facebook and Twitter accounts), and notices were sent to those individuals with BD who had been recruited for previous studies for whom we had ethical permission to contact. Participants were free to self-select which of the 4 KT strategies they wanted to engage with. Participants were remunerated with a Can $10 gift certificate to recognize their time in the webinar or video (quantitative) arms, Can $20 for participation in the workshop or Living Library (quantitative) arms, and an additional Can $20 for participation in the qualitative arm.

### Quantitative Methods, Statistical Analysis, and Assessment Scales

Ethics approval for the study was granted by the University of British Columbia’s Behavior Research Ethics Board. All participants received written information on the study and gave written informed consent. Participants consenting to the quantitative arm of the study were given the option of consenting to be re-contacted for a qualitative interview.

Quantitative assessments of the experience and impact of the KT strategies were conducted in 2 ways. First, we investigated the subjective experience of participating in the KT strategy immediately upon completion of contact (“immediate posttest”). Participants rated each of the 4 statements related to a particular KT strategy (ie, “learned something new,” “applicable to me,” “met my expectations,” and “would recommend to others with BD”) on a Likert scale from Strongly Agree (1) to Strongly Disagree (5). Second, we investigated the short-term impact of the KT strategy by comparing 3-week follow-up ratings (Time 2) with baseline scores (preintervention, in the week before engagement with the KT strategy [Time 1]). Only a 3-week assessment time frame was feasible for this exploratory project. Quantitative assessment of the impact of the KT strategies on health outcomes utilized 4 scales, assessing QoL (via the 56-item QoL.BD, a condition-specific QoL scale, which is sensitive to change in clinical state over 7-10 days [25]); recovery (via the 36-item Bipolar Recovery Questionnaire (BRQ) [29]); mood (via the Positive and Negative Affect Schedule [PANAS], a self-report questionnaire that consists of two 10-item scales to measure both positive affect (PA) and negative affect (NA) [30]); and Chronic Disease Self-Efficacy (SECD, via the 5-item “Manage Disease in General” subscale of Stanford’s Chronic Disease Self-Efficacy Scales [31,32]). Quantitative data were analyzed using the Statistical Package for the Social Science (software) version 24.

By necessity, the data collection approach differed slightly by the KT arm. In the webinar arm, research participants self-selected a single webinar to participate in based on their own interests; webinars were scheduled at a rate of approximately 3 per week from April to May 2015. A week before the webinar, research participants were sent a unique link to the Time 1 assessments. After participating in the webinar and Q&A, they were sent a link to the immediate posttest questions, and 3 weeks after that, another link to the Time 2 assessments. In the video arm, the videos were embedded directly into the Qualtrics system; participants conducted the Time 1 assessments, viewed the video, immediately conducted the immediate posttest questions, and were sent a link to the Time 3 assessments 3 weeks later. In the Living Library arm, participants were sent a link to the Time 1 a week before their scheduled Living Library session, sent a link to the immediate posttest questions on completion of the session, and then a link to the Time 2 assessments 3 weeks later. Finally, for the workshops, delivered in May to June 2015, the Time 1 and 2 assessments were administered via Qualtrics, but the immediate posttest questions were completed by paper and pencil at the end of the workshop.

### Qualitative Methods and Data Analysis

Purposeful criterion sampling [33] was used to ensure the breadth of KT modalities was represented. Consenting participants were contacted via email approximately 2 weeks after participating in the KT strategy to schedule a telephone interview; interviews occurred approximately 3 to 4 weeks following participation in the KT strategy. A total of 43 interviews were conducted by coauthor EM. Interviews ranged from 20 to 70 min in length (mean 39.4, SD 11.2). A semistructured interview schedule was developed with 3 main sets of open-ended questions. The first set of questions asked participants about their engagement with the information shared in the KT session. The second set of questions explored any attempts to implement self-management strategies. Finally, participants were asked about their QoL, including any changes to their QoL following exposure. Probes and reflective listening were used to elicit depth in participant responses. The interviews were digitally recorded and transcribed verbatim by EM (24/43, 56%) or research assistants (checked by EM for accuracy; 19/43, 44%).

Thematic analysis [34] of participants’ perceptions and responses to the content and delivery of the self-management information in the KT strategy occurred. Themes describing participants’ experiences of engaging with a KT strategy are discussed in detail elsewhere [10]. For the purposes of this paper, a secondary analysis was conducted that focused on information that would add to or clarify understanding of the quantitative study results. Step 3 of Braun and Clarke’s [34] analytic process informed the analysis producing *descriptive, literal categories* of the data. Examples of positive and negative experiences relating to the KT modalities were identified. Data were assigned brief descriptive codes in the qualitative data management software NVivo [35]. Codes were examined, and overarching categories were identified. The content of categories was reviewed for coherency, and transcripts were revisited as the themes were developed to ensure all relevant data were adequately described. The essence of the most important categories in relation to understanding quantitative data was then described in a report with illustrative transcript extracts. To reduce the risk of bias and to address analytic validity, authors EEM and RH reviewed both the descriptive accounts of themes and transcripts for coherency and validity of interpretation, with disagreements resolved via consensus.

## Results

### Participants

A total of 94 participants were included in the quantitative analyses, with a modal age range of 45 to 54 years and the majority (n=53, 56%) identifying as female. The most frequently reported diagnosis was BD Type I (46/94, 49%), followed by BD-II (31/94, 33%). In the 43 participants in the qualitative analyses, modal age range was 45 to 54 years; n=30 (70%) participants were female, with n=24 (56%) reporting BD Type I and 16 (37%) reporting BD Type II.

### Quantitative Results

We first analyzed the immediate postintervention data to explore participants’ perceptions of the different types of KT strategies. Data were analyzed using a one-sample *t* test comparing the difference between the mean response and the neutral rating of 3. As shown in Table 1, participants were very positive about all KT strategies with modal response of Agree or Strongly Agree in each case and responses significantly better than neutral for 15 of the 16 cells.

To understand the impact of participation in the 4 KT strategies over time, we first conducted a multivariate repeated-measures (pretest to follow-up on each of BRQ, QoL.BD, PA, NA, and SECD) analysis of variance with KT strategy (ie, webinar, video, Living Library, and workshop) as the between-subjects variable. The analysis revealed a main effect of Time: when averaged across the 4 strategies, participation led to improvements across the 5 outcomes (upper 5 rows of Table 2). Posthoc analyses found nonsignificant improvements in PA and NA and significant improvement as measured on the BRQ (*P*=.02) and QoL.BD (*P*=.005). Chronic Disease Self-Efficacy as measured by SECD was the outlier, with a small (nonsignificant) decrease when averaged across the 4 strategies.

**Figure 7:**
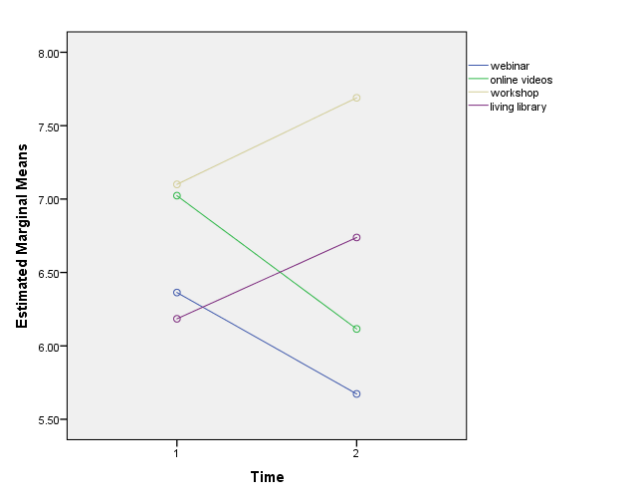
Pretest to follow-up change in chronic disease self-efficacy by strategy (difference in slopes *P*=.01).

Second, we looked at the 5 outcome variables separately, via a Time × Strategy interaction (bottom 5 rows of Table 2). For BRQ, QoL.BD, PA, and NA, there was no difference between the KT strategies in terms of their ability to improve outcomes (ie, there was no significant Time X Strategy effect for these 4 variables). Interestingly, the data showed that for SECD, there was a significant Time ×Strategy effect (*P*=.01; the sole outcome that showed a negative average impact across strategies). Figure 7 shows that SECD declined in the 2 conditions that were more technology-dependent (webinar and video), but actually improved in the 2 conditions that had more peer or interpersonal contact (Living Library and workshop).

**Table 1:** Ratings of satisfaction with 4 knowledge translation strategies (1=Strongly Agree and 5=Strongly Disagree). Data in the table are mean (mode) responses. Superscripted letters refer to the significance of one-sample *t* test comparing mean with neutral response (ie, rating of 3).

Strategy	Something new, mean (SD)	Applicable, mean (SD)	Expectations, mean (SD)	Recommend to others with bipolar disorder, mean (SD)
Webinar (n=22)	2.41 (2)^a^	1.77 (1)^b^	2.55 (1)	1.77 (1)^b^
Video (n=26)	2.35 (2)^a^	1.85 (2)^b^	2.50 (2)^c^	1.65 (2)^b^
Workshop (n=32)	1.78 (2)^b^	1.34(2)^b^	1.81 (2)^b^	1.56 (2)^b^
Living Library (n=14)	1.93 (2)^b^	1.86 (1)^b^	2.21 (1)^c^	1.43 (1)^b^
Combined (n=94)	2.11 (2)^b^	1.66 (1)^b^	2.23 (2)^b^	1.62 (1)^b^

^a^*P<*.005.

^b^*P*<.001 (survives Bonferroni adjustment for 16 comparisons).

^a^*P<*.05.

**Table 2:** Results of multivariate analysis of variance, testing change in 5 outcome measures between pretest and follow-up (upper 5 rows are main effect of time and lower 5 rows are the interaction between time and knowledge translation strategy).

Analysis and measure		Mean square	F *(df)*	*P* value
**Time**				
	Bipolar Recovery Questionnaire	205.053	5.887 (1,77)	.02
	Quality of Life in Bipolar Disorder (scale)	1.252	8.212 (1,77)	.005
	Positive Affect^a^	0.457	1.889 (1,77)	.17
	Negative Affect^a^	1.236	3.059 (1,77)	.08
	Chronic Disease Self-Efficacy	0.490	0.283 (1,77)	.60
**Time X Strategy**				
	Bipolar Recovery Questionnaire	23.241	0.667 (3,77)	.58
	Quality of Life in Bipolar Disorder (scale)	0.302	1.982 (3,77)	.12
	Positive Affect^a^	0.091	.376 (3,77)	.77
	Negative Affect^a^	0.404	0.999 (3,77)	.40
	Chronic Disease Self-Efficacy	6.339	3.652 (3,77)	.02

^a^A measure from the PANAS scale.

### Qualitative Results

Participant descriptions of both positive and negative experiences specifically associated with the webinar, video, Living Library, and workshop arms are summarized in Tables 3-6, respectively. Positive aspects of the various KT strategies emphasized by participants included the interactive nature of the webinars and the depth of information provided, and the brevity and visual accessibility of the videos. Across the KT modalities, peer contact was consistently emphasized as a helpful or appreciated aspect, but descriptions of how peer contact was experienced varied by modality. Participants in the workshop arm emphasized appreciating the opportunity to learn from others managing the same condition and reducing self-stigma though normalization of shared experiences. The Living Library was described as a helpful place to share experiences with an expert who could understand and relate to them, and some participants described being inspired by having access to somebody who lived well with BD. Participants in the video arm often felt able to relate to the actor or narrator, which helped them better understand their own experiences as well as identify the personal relevance of strategies.

**Table 3:** Qualitative findings: positive and negative experiences of webinars.

Category description		Quote
**Positive experiences**		
	Interactive (n=7)	“I also really liked that they had a question and answer period at the end.” [F, BD-II, 25-34]
	Depth of information (n=5)	“I find them very informative, and I personally like webinars.” [F, BD-I, 55-64]
	Peer presence as normalizing or providing community (n=3)	“I saw the people on the right, the people who had signed in, and the comments. So it just said, ‘Well, you’re in good company, and you can do this, and you’re doing things right,’ kind of.” [F, BD-I, 55-64]
**Negative experiences**		
	Overly academic or clinical (n=4)	“What kind of triggered me at the beginning was a lot of what I call MBA type language...I find that really, really frustrating, because the emphasis on quality indicators and efficacy and all this kind of stuff, really is never about the person.” [M, BD-NOS, 45-54]
	Technical difficulties (n=1)	“Well for one thing I entered the webinar rather late because I was having some system problems, so I had to contact the webinar organisers and get everything sorted out. So I might not have learned as much as I could have.” [F, BD-I, 55-64]

**Table 4:** Qualitative findings: positive and negative experiences of videos.

Category description		Quote
**Positive experiences**		
	Succinct (n=3)	“It’s not overwhelming, it’s not like reading a thousand page book, the videos are short and sweet and I think that’s good.” [M, BD-I, 55-64]
	Visualizing self-management (n=3)	“Watching somebody do things...that’s how I learn, and I found the video is really helpful in that...It gave me a sort of visual concept of what it’s about.” [F, BD-I, 55-64]
	Relatability of the narrator (n=5)	“Just seeing how that video was, like first of all, how her life was with her depression or how she was feeling, her mood, how things were cluttering her world, I recognize that that’s what happens to me, but to know it, to actually see it was helpful.” [F, BD-II, 45-54]
**Negative experiences**		
	Difficulty identifying with a person living well with bipolar disorder (n=4)	“I didn’t’ connect with the video as much as I would have liked to, because I felt like a bit of a personal failure because it wasn’t that easy for me, it was so much harder.” [F, BD-II, 45-54}
	Amount of information (n=2)	“I found that the videos were very short and not very, from a user’s point of view, not that informative.” [M, BD-I, 35-44]

**Table 5:** Qualitative findings: positive and negative experiences of Living Library.

Category description		Quote
**Positive experiences**		
	Living Library expert understands my experiences (n=2)	“One of strongest points of our talk was finding out that [the Living Library expert] was also bipolar. And that’s what I’ve been searching for, is to talk to others that live it, and not just read about it in a book, because even then, when I read some of the books, it doesn’t quite fit what I’ve experienced and maybe I’m looking for others like me” [F, BD-I, 45-54]
	Being inspired by the Living Library expert (n=4)	“Knowing that people go through the same stuff and probably manage the condition better. So just knowing that helped. *”* [M, BD-I, 25-34]
**Negative experience**		
	Lack of information provided by the Living Library expert (n=1)	“I learned absolutely nothing...She didn’t understand the program herself, she couldn’t help me get into some of the stuff.” [F, BD-I, 55-64]

**Table 6:** Qualitative findings: positive and negative experiences of workshops.

Category description		Quote
**Positive experiences**		
	Learning what works for others with bipolar disorder (n=5)	“I found that was also helpful, sharing with other people, you know suggestions about things that have helped them and so on.” [M, BD-II, 35-44]
	Normalizing difficulties (n=4)	“I felt when I was there that I didn’t stand out. It was just kinda like everybody was there and everybody has their own little struggles.” [F, BD-I, 35-44]
**Negative experience**		
	Scheduling (n=1)	“I would have preferred if the workshops had been a bit earlier, especially because we ended up going later. I found that hard, you know, we’re talking about sleep - we’re talking about going to bed at the regular, you know, for setting up the pattern. And I ended up going to bed late that night. So that was, that was a bit frustrating, to be talking about setting up a regular sleep time and then, set back, from doing that, rolling my sleep schedule off.” [F, BD-I, 35-44]

Reports of negative experiences of KT modalities were infrequent, particularly in participant descriptions of the workshop and Living Library arms (2 participants across both these arms described unique negative experiences). As such, description of negative experiences in this section focuses on the webinar and video arms, where a small number of participants described feeling disengaged or discouraged by the interventions. Overall, 4 participants in the webinar arm described the academic style of information delivery in the webinar as off-putting and 1 reported technical difficulties. Some participants found it difficult to relate to the depiction of a woman living well with BD in the videos, resulting in a sense of discouragement or frustration. Although the length of the videos was described by some participants as a positive, others found that their brevity limited the amount of useful information conveyed. Finally, of note, a preference for in-person services was described by some participants across both the webinar and video arms.

## Discussion

### Overview

The potential for digital technologies to produce a transformative impact in the mental health arena is profound. Research is burgeoning in this space, driven in part by recognition of the remarkable rate at which society is adopting technology into their everyday lives, the pressures on health care systems for services to be delivered flexibly in a patient-centered manner, and acknowledgment of the empowerment that digital technologies can bring to patients by enabling them to make choices about when and how they access care [36]. Technology-supported self-management holds significant potential to improve the health and QoL of people facing mental health challenges and represents 1 avenue to reduce the substantial health care costs associated with chronic illnesses [37]. However, the production of evidence-based self-management support technologies is time and resource intensive and requires sustained maintenance; the identification of effective approaches to optimize user engagement with them is therefore paramount. This mixed-methods study explored the impact of 4 KT strategies on user engagement and health-related outcomes in people with BD.

### Principal Findings

Quantitatively, at a broad level, participants evaluated all 4 KT strategies positively. When the KT strategies were examined collectively, we observed a nonsignificant trend toward increased PA and decreased NA following exposure to the KT strategies and significant improvements in perceived recovery and QoL. A modest nonsignificant decrease in perceived self-efficacy to manage chronic illness was observed after exposure. Exploration of the impact of the KT strategies individually indicated that all 4 KT strategies positively impacted perceived recovery, QoL, and both PA and NA. Chronic disease self-efficacy was observed to significantly decline in the more technology-dependent conditions (webinar and video) but improve in the 2 conditions with more peer and interpersonal contact (workshop and Living Library).

This study adds one piece of incremental evidence to a growing literature, suggesting that Web-based technologies can represent an effective conduit for, or complement to, care for people with BD. In a recent systematic review of Web and mHealth apps for self-management in BD, for example, we identified 15 studies, several of which demonstrated positive impacts of digital interventions on QoL [11]. The most commonly supported self-management strategy categories delivered by Web-based interventions identified in the review were “ongoing monitoring,” “maintaining hope,” “education,” and “planning for and taking action”; the least commonly supported categories were “relaxation” and “maintaining a healthy lifestyle.”

Qualitatively, user experiences of the 4 KT strategies tested were also largely positive. Most dominant was an appreciation of the opportunity for peer contact. Negative descriptions of experiences tended to focus on the webinar and video strategies, citing issues such as disliking the academic tone of the webinars, or an inability to identify with the woman living well with BD depicted in the videos. Broadly speaking, our qualitative findings suggested that there was no “one size fits all” approach for determining the right type of KT strategy for BD self-management information. Other qualitative analyses of this qualitative dataset have been published, with a focus on participants’ experiences of self-management more broadly as well as perception of their QoL [10,38]. Although this analysis focused on perceptions of the KT strategies embedded in the overall self-management intervention, an evaluation of experiences of engaging with the self-management intervention more broadly as well as attempts to enact self-management strategies was conducted [10]. Feelings of empowerment and responsibility to self-manage BD were discussed, whereas a minority of participants felt that self-management strategies lacked power to control BD symptoms. The relationship of self-management to the health care system was also discussed, with opportunities to develop a sense of partnership between clinicians and patients via attention to self-management identified. A more in-depth discussion of the links of such experiences to self-efficacy, recovery, and therapeutic alliance is presented therein. In addition, a qualitative analysis focusing on individuals’ subjective experiences of their QoL was conducted [38]. The relativity of subjective QoL judgments to self, others, and possible futures was explored, and the possible implications of the flexibility of reference point usage for future therapeutic interventions were discussed.

Our observation that chronic disease self-efficacy may be differentially impacted by the mode of KT strategy (ie, ones containing more vs less peer interaction) is intriguing. On the one hand, existing literature clearly points to the potential benefits of Web-based peer support in terms of increasing feelings of social connectedness, group belonging, and serving as an avenue to share self-care strategies [39]. Web-based peer support for people with mental health problems can occur in diverse ways, including via general support forums (eg, [40]); advanced interactive forums dedicated to sharing illness experiences such as PatientsLikeMe [41]; discussion forums that are embedded within formal Web-based treatment interventions (as being evaluated, eg, in the BD Mood Swings 2.0 [42,43] and ORBIT [44] trials); or through social media platforms such as Twitter, Facebook, and Reddit [39,45] and blogging [46] or video-based platforms [47,48]. Regardless of the conduit, it appears that engagement rates with apps that foster social connections may be higher [49].

Web-based engagement should not be viewed, however, as entirely without risk; Naslund et al [39] note that interactions on the Web can result in exposure to unreliable information, hostile or derogatory comments, or feelings of uncertainty or anxiety about one’s mental health condition. Our finding, albeit from a modest dataset, that chronic disease self-efficacy may be negatively impacted by KT strategies with less peer support may be 1 example of how digital interventions might also have unintended negative consequences. In a recent study, Williams et al [47] qualitatively explored users’ experiences of lived experience videos on an interactive recovery-oriented website (the Self-Management and Recovery Technology or “SMART” program) designed for people with psychosis. Although the delivery and focus of the videos in the SMART program differed from those of this study (SMART program videos were focused on recovery principles rather than on self-management strategies and were designed to be viewed either with a mental health worker or independently), results from that study are germane to this project. In particular, participants’ experiences of the videos were clearly situated for many in the context of their personal recovery journey, as the authors’ state:

Responses to the videos appeared to be influenced by the participants’ existing ways of coping with life and managing their recovery, as well as by how they used technology. Participants who did not relate to the peers as role models perceived that their ways of coping were too different, or they were at a point in their recovery journey where they did not want to identify with peers.

In summary, our quantitative (ie, decreased chronic disease self-efficacy scores in the video and webinar arms) and qualitative (ie, some participants reporting difficulties identifying with the actor living well with BD in the videos) findings suggest 2 things. First, participants’ experiences of KT strategies appear to be influenced by their understandings of their position in their own recovery journey. Second, there appears to be benefit of KT strategies that incorporate peer support. Future research is needed to more fully explore the precise relationship between peer support and engagement with, or enactment of, concrete self-management strategies.

### Limitations

A number of limitations to this study should be noted. First, participants self-reported their diagnosis of BD; although confirmation of diagnosis by structured psychiatric interview would have been preferable, there is some evidence that people who self-identify as living with BD typically do meet diagnostic criteria [50]. Second, this study was positioned as an exploratory phase in a longer-term program of research to advance understanding of the utility of digital health technologies for supporting self-management in people with BD. Participants were volunteers who were paid a modest honorarium for their participation and were free to self-select which KT modality they wanted to engage with, likely biasing toward an engagement method they were intrinsically attracted to, which in turn may have impacted the range of positive and negative experiences reported, limiting the generalizability of the results to routine health service delivery settings. Third, the assessment time frame for the examination of quantitative outcomes was short, that is, 3 weeks. Many digital health intervention studies apply assessment periods of 4 to 8 weeks (eg, [51]). Furthermore, changes in the outcomes of interest in this study, such as QoL, are anticipated to emerge relatively slowly in BD compared with changes in symptoms (eg, [52]), and changes in self-management behaviors are fully expected to take time to incorporate into everyday life. Longer assessment time frames in future work are called for to fully capture expected trajectories of change in QoL outcomes and self-management behaviors. Unlike QoL, a sense of personal recovery as measured on BRQ might be expected to shift quite quickly as people adopt new attitudes toward their role in managing BD. Responses to BRQ items such as “I have the resources to manage my health” and “The activities I do make a difference to others” could shift solely via insights gained from interactions in the intervention. Fourth, as there was no control group of participants who did not partake in any strategy, nonspecific causes (such as attention, time, demand characteristics, or the honorarium paid for participation) cannot be ruled out as an explanation for the observed quantitative outcomes.

### Implications and Future Directions

Despite the aforementioned limitations, this study provides a useful mixed-methods evaluation of the potential impact of a range of KT strategies on health-related outcomes in people with BD. The study was conducted between 2014 and 2016; our CREST.BD program of research has been focused since then on meeting emerging opportunities and needs of the digital mental health landscape. Moving forward, we are seeking funding for work to incorporate the evidence and tools held in the Bipolar Wellness Centre [23], our Web-based QoL Tool [26], and the findings from this study to inform the development of a new mHealth app—“Bipolar Bridges.” In doing so, we are carefully addressing some of the key challenges currently being faced by the digital-technology space, which relate prominently to issues of information credibility, safety, and privacy.

More than 10,000 mental health apps are available for immediate download today; worryingly, most mental health apps in commercial marketplaces are not evidence-based nor conform to clinical guidelines [53]. A 2015 systematic review found the majority of apps for BD (82/571 available apps were reviewed) were not aligned with clinical guidelines or established self-management principles and did not cite sources of evidence [54]. Some may even offer dangerous recommendations (eg, 1 app recommends that people experiencing BD mania drink alcohol at bedtime to assist with sleep [54]). Furthermore, many available apps do not respect the privacy of personal health information [53-55]. Thus, there are significant barriers for people facing mental health challenges in identifying safe, evidence-informed apps [56,57]. Data from adults with BD [58] report “wish list features” for new apps inclusive of “social interaction capability” and “between-app integration” and receptiveness to digitally supported self-management [59]. Data from youth with BD report prioritization of ease-of-use, scientific quality, customization, and data privacy [16]. At a broad level, the BD field needs to advance in terms of creating technology-supported spaces, which are inclusive of diverse people, regardless of their gender, sexual orientation, socioeconomic status, digital literacy, geography, and ethnicity. Finally, the results of this study indicate that more work is needed to inform how to best advance social connectivity via apps. Looking ahead, our Bipolar Bridges project aims to address some of these key challenges via the creation of a new app designed to enable diverse users with BD to optimize their QoL via supported self-management, resting on an innovative and secure blockchain-based platform. The results of this study will be applied to hone a comprehensive mixed-methods (quantitative, qualitative, and machine learning) investigation of the feasibility, engagement, adoption, impact, and mechanisms of change associated with the use of the app.

## Abbreviations

BD: bipolar disorder
BRQ: Bipolar Recovery Questionnaire
CBPR: community-based participatory research
CIHR: Canadian Institutes of Health Research
CREST.BD: Collaborative Research Team to study psychosocial issues in Bipolar Disorder
KT: knowledge translation
MDAO: Mood Disorders Association of Ontario
mHealth: mobile health
NA: negative affect
PA: positive affect
PANAS: Positive and Negative Affect Schedule
Q&A: questions and answers
QoL.BD: Quality of Life in Bipolar Disorder
QoL: quality of life
